# What was the impact of COVID-19 restrictions on unintentional injuries, in Canada and globally? A scoping review investigating how lockdown measures impacted the global burden of unintentional injury

**DOI:** 10.3389/fpubh.2024.1385452

**Published:** 2024-06-03

**Authors:** Shazya Karmali, Shikha Saxena, Olivia Richards, Wendy Thompson, Steven R. McFaull, Ian Pike

**Affiliations:** ^1^BC Injury Research and Prevention Unit, BC Children’s Hospital Research Institute, Vancouver, BC, Canada; ^2^Public Health Agency of Canada, Ottawa, ON, Canada; ^3^Faculty of Medicine, Department of Pediatrics, University of British Columbia, Vancouver, BC, Canada

**Keywords:** pandemic (COVID19), public health measure, accident, lockdown, restriction

## Abstract

**Background:**

Injuries are among the leading causes for hospitalizations and emergency department (ED) visits. COVID-19 restrictions ensured safety to Canadians, but also negatively impacted health outcomes, including increasing rates of certain injuries. These differences in trends have been reported internationally however the evidence is scattered and needs to be better understood to identify opportunities for public education and to prepare for future outbreaks.

**Objective:**

A scoping review was conducted to synthesize evidence regarding the impact of COVID-19 restrictions on unintentional injuries in Canada, compared to other countries.

**Methods:**

Studies investigating unintentional injuries among all ages during COVID-19 from any country, published in English between December 2019 and July 2021, were included. Intentional injuries and/or previous pandemics were excluded. Four databases were searched (MEDLINE, Embase, Web of Science, SPORTDiscus), and a gray literature search was also conducted.

**Results:**

The search yielded 3,041 results, and 189 articles were selected for extraction. A total of 41 reports were included from the gray literature search. Final studies included research from: Europe (*n* = 85); North America (*n* = 44); Asia (*n* = 32); Oceania (*n* = 12); Africa (*n* = 8); South America (*n* = 4); and multi-country (*n* = 4). Most studies reported higher occurrence of injuries/trauma among males, and the average age across studies was 46 years. The following mechanisms of injury were reported on most frequently: motor vehicle collisions (MVCs; *n* = 134), falls (*n* = 104), sports/recreation (*n* = 65), non-motorized vehicle (*n* = 31), and occupational (*n* = 24). Injuries occurring at home (e.g., gardening, home improvement projects) increased, and injuries occurring at schools, workplaces, and public spaces decreased. Overall, decreases were observed in occupational injuries and those resulting from sport/recreation, pedestrian-related, and crush/trap incidents. Decreases were also seen in MVCs and burns, however the severity of injury from these causes increased during the pandemic period. Increases were observed in poisonings, non-motorized vehicle collisions, lacerations, drownings, trampoline injuries; and, foreign body ingestions.

**Implications:**

Findings from this review can inform interventions and policies to identify gaps in public education, promote safety within the home, and decrease the negative impact of future stay-at-home measures on unintentional injury among Canadians and populations worldwide.

## Background

On March 11, 2020, the rapid spread of coronavirus SARS-CoV-2–a highly contagious virus causing flu-like symptoms and potentially hospitalizations and death–resulted in the declaration of the COVID-19 pandemic by the World Health Organization (1). This was followed by countries worldwide rapidly administering public health policies to limit and reduce the spread of the virus, including stay-at-home and physical distancing measures. In Canada, provinces began implementing COVID-19 policies in early March 2020: workplaces shifted to work-at-home arrangements, schools employed virtual learning, and restaurants, recreational spaces, and other “non-essential” businesses closed their doors (2). Despite restrictions to keep populations safe from the virus, injuries continued to occur during this time, including those related to falls, transportation, physical activity, drowning, suffocation, poisoning, burns, violence, self-harm, and suicide (3).

Injuries are one of the leading causes of visits to doctors’ offices, emergency departments, and hospital admissions for all age groups, the most common cause of death in Canadians aged 1–34 years, and the sixth leading cause of death among all ages combined (3–5). Unintentional injuries account for the majority of injury cases, causing 75% of deaths, 89% of hospitalizations, 95% of emergency department visits, and 90% of disabilities (6). Additionally, falls and poisonings were the two leading causes of injury deaths in Canada in 2015 (5). Injuries are predictable and preventable; however, policies enforcing stay-at-home measures, distanced activities, and the disruption of daily routines, had an unknown impact on unintentional injuries.

Prior to the COVID-19 pandemic, researchers suggested the development of specific critical care service and ethical considerations during pandemics and disasters (7, 8). They noted that during pandemics or disasters, health care facilities may experience a surge in patients, and vulnerable populations may be further marginalized due to the challenges in accessing health care services (7, 8). To address these issues, Hick and colleagues (8) created recommendations through a modified Delphi process for those involved in large-scale disaster and pandemic responses, particularly with respect to critically ill or injured patients. Their recommendations included capacity and capability planning for mass critical care, increasing awareness and information sharing, reducing the burden on critical care facilities, planning for the care of vulnerable populations, and the reallocation of healthcare resources (8). The recommendations were then categorized to those that were most relevant to each of their target audiences (i.e., clinicals, hospital administrators, and public health/government). Using a similar approach to explore and understand the most common mechanisms of injury during disaster, pandemic, and lockdown periods will allow researchers, healthcare providers, and public health and government officials to better identify gaps in healthcare response and capacity. In addition, recognizing the most common mechanisms of injury and high-risk settings during disasters or pandemics will allow for the development of focused public education and awareness campaigns, facilitating the reduction of injury incidence during and outside of lockdown periods.

The COVID-19 pandemic affected populations at a global level, and its impact on hospitalizations and emergency department visits varied at provincial and national levels from country to country. The primary reason for this variation could potentially be due to the dissimilarities in how and when the public health measures were implemented. A large body of evidence has been published by different countries across the globe comparing the trends and patterns of unintentional injuries during COVID-19 versus pre-pandemic. From a public health and policy perspective, it is essential to synthesize and understand the similarities and differences in the trends of these unintentional and potentially preventable injuries, so that injury burden and the utilization of health care services and resources may be reduced during future similar circumstances.

The COVID-19 pandemic highlighted the importance of reliable and comprehensive data that would allow for informed decision-making. The Public Health Agency of Canada is responsible for the national surveillance of intentional and unintentional injury and poisoning (9). Unfortunately, routine data collection for many national data sources was impacted by the pandemic and data collection reduced/ceased. Thus, this review also aimed to identify how other health data continued to be collected during the pandemic, and how similar methods might be implemented and utilized to assess injury patterns in Canadian systems in future events. The objective of this review was to – (a) explore and summarize evidence supporting the effects of public health measures related to COVID-19 on the trends and patterns of unintentional injuries in Canada; (b) to compare the trends and patterns of unintentional injuries in Canada with those observed in other similar countries.

## Method

This scoping review was conducted according to the methodological framework designed by Arksey and O’Malley (10), and consisted of five stages: (1) identifying the research question; (2) identifying relevant studies; (3) study selection; (4) charting the data; and (5) collating, summarizing, and reporting the results. The research team included experts in injury epidemiology, evidence synthesis, disability, injury rehabilitation and knowledge translation. Through a series of iterations, the research team agreed upon the scope of the review, inclusion criteria and extraction variables.

In consultation with an academic librarian, Ovid MEDLINE, Embase, Web of Science Core Collection, and SPORTDiscus were searched for scholarly articles for the period December 2019 to July 2021, inclusive. The search strategy consisted of injury-related terms, including wounds and injuries, burns, drowning, electric injuries, occupational injuries, fractures, etc. A Google search for gray literature was also conducted, in which the first 50 results for each mechanism of injury were extracted and screened. The full search strategy is documented in Appendix A (11).

### Research questions

The research questions that guided this scoping review, and ensured that a wide range of literature was captured were: (a) What were the effects of the public health measures related to COVID-19 on trends and patterns of unintentional injuries in Canada? (b) How are these trends and patterns comparable to other similar countries?

### Identifying relevant studies

To ensure the comprehensiveness of this scoping review, we searched four electronic databases – (MEDLINE, Embase, Web of Science, SPORTDiscus). We also included gray literature, including theses/dissertations and reports revealed by Google. Studies were included if they were published in English, within the indicated time frame and addressed unintentional injuries among all age-groups and all countries, worldwide. For the purposes of this review, unintentional injuries were defined as any injury that is not caused on purpose or with intention to harm (11). Studies were excluded if they focused on intentional injuries and/or previous pandemics. Only primary research was included in the final article selection (i.e., no reviews, letters to the editor, etc.). With respect to gray literature searches, records were selected for inclusion if they were published by news outlets, blogs, non-profit organizations, academic institutions, government, or public health websites.

### Study selection

The identification of the search strategy followed an iterative process. First, we conducted a preliminary search in Medline and explored article titles, abstracts, keywords and subject headings to develop our search strategy. We then included the identified keywords and subject headings in the search strategy of all four databases, constituting the final search strategy. A faculty librarian provided expert guidance and verification regarding the appropriate subject headings, and adaptation of search strategies across databases. Search results were imported into Covidence (12) and duplicates were automatically removed by the software. To ensure accuracy in screening and capturing relevant studies using the search criteria in Covidence, three reviewers (SK, SS, OR) screened a random sample of 50 articles. During the first round of screening (title and abstracts), the reviewers erred on inclusion and an iterative process involving meetings among the reviewers to refine search criteria was used. Title and abstracts followed by full-text review were completed independently by two reviewers (SK, SS or OR) using Covidence. For disagreements among the reviewers, data from the article was discussed to achieve consensus. If consensus could not be reached, a third reviewer was consulted and served as arbiter to reach consensus.

### Charting the data

Once the final studies were selected, data extraction was conducted using Microsoft Excel. Data was extracted based on the following categories: fell within the date range for data collection, study design, data sources, sample size, demographics, and injury descriptors. The injury categories could not be mutually exclusive as their classification varied across regions. The categories included the external causes of injuries (falls, non-motorized vehicle collisions, trampoline, motor vehicle collisions, sports/recreation; pedestrian vs. motor vehicle, burns, work-related, crush/trapped, lacerations, foreign body ingestion, poisoning, drowning), and injury settings (e.g., at home, at work, etc.).

## Results

### Search results

**Figure 1:**
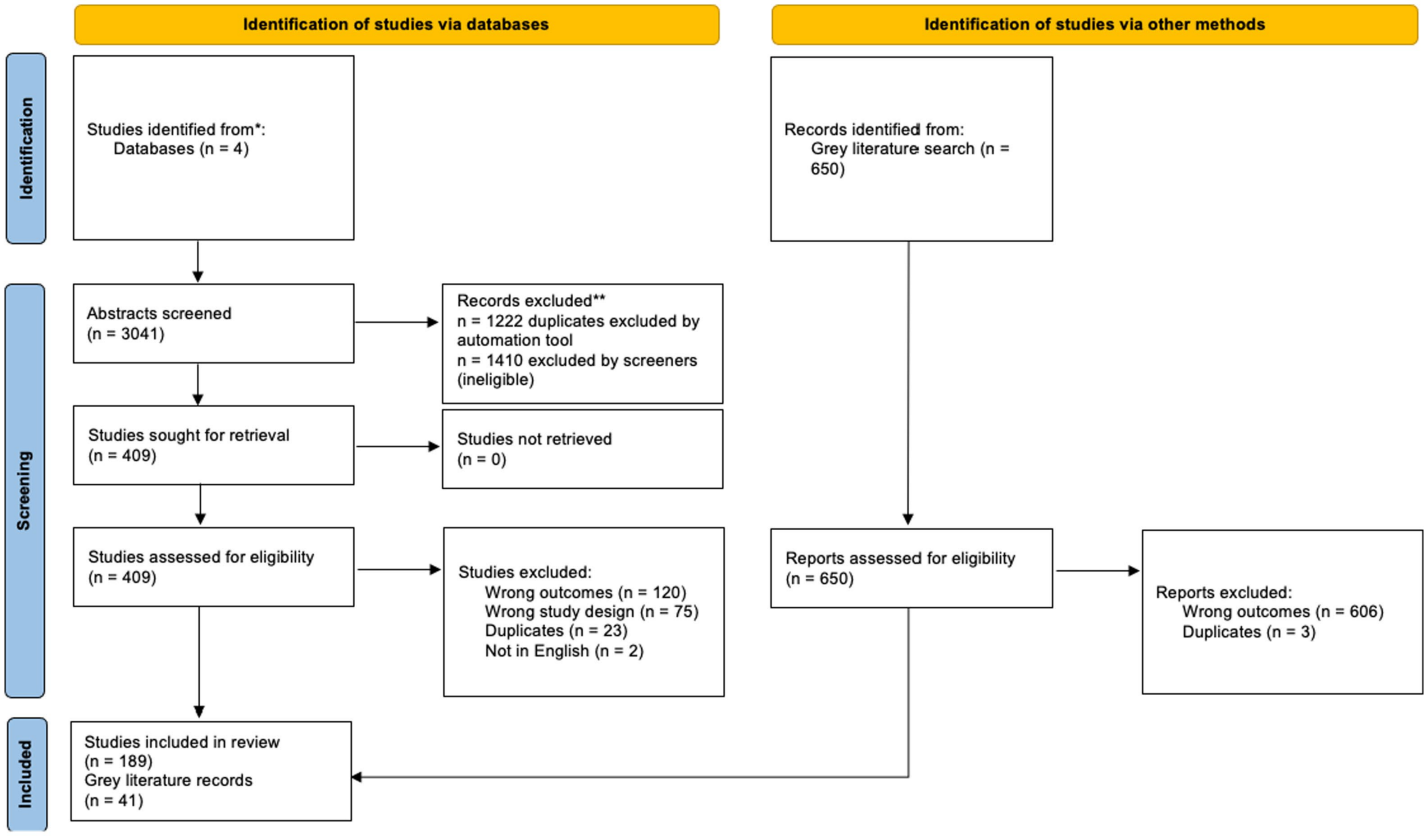
PRISMA table of articles selected for study.

Of the 3,041 references uploaded to Covidence, 1,222 were duplicates, 1,819 were screened for title and abstract relevance, and 409 were advanced to full-text review. A total of *n* = 189 peer-reviewed articles were included in final analysis. An additional *n* = 41 gray literature records were also included. See Figure 1. PRISMA table outlining study selection process.

### Study characteristics

Most studies compared pandemic period trends in their respective countries to the same dates during pre-pandemic time periods to explore differences in injury presentations/cases. The pandemic time periods varied depending on the region, and the pre-pandemic periods included between one and 5 years prior to COVID-19 lockdowns. For an overview of study characteristics, including region, study design, and mechanism of injuries see Table 1.

**Table 1:** Overview of study characteristics.

Region	*n*, %
Europe	85, 45.0%
North America	44, 23.3%
Asia	32, 16.9%
Oceania	12, 6.3%
Africa	8, 4.2%
South America	4, 2.1%
Multi-country	4, 2.1%
Study design	
Retrospective review of databases	128, 67.4%
Cohort	23, 12.1%
Other (Time-series, comparative retrospective, secondary analysis, prospective review, quasi-experimental)	18, 9.5%
Cross-sectional	16, 8.4%
Case control	5, 2.6%
Injury categories	
Motor vehicle collision/motorcycle collision	134, 71.0%
Falls	104, 55.0%
Sports/recreation	65, 34.4%
Non-motorized vehicle	31, 16.4%
Occupational injury	24, 12.7%
Unintentional injury**	21, 11.1%
Pedestrian injuries	20, 10.6%
Laceration (including bites)	18, 9.5%
Burns	17; 9.0%
Poisoning	10; 5.3%
Crush/trapped	6; 3.2%
Trampoline	6; 3.2%
Foreign body ingestion	2, 1.1%
Drowning	1*

*Negligible percentage. **No injury type specified.

Notably, only three studies that met inclusion criteria were based in Canada at the time of the search, and another study was conducted in both the United States and Canada. A total of 144 (76%) of the included articles presented findings on injuries occurring in all age groups, whereas 22 (11.6%) reported on pediatric cases only, 25 (13%) on adult, and one on older adults. In addition, many articles reported on more than one mechanism of injury. A total of 111 (59%) studies indicated that injury incidence was higher among males than females, and the average age across all studies was 45.7 years old (Range or SD?). Various data sources were employed to collect data for each of the studies, with the most frequently used being electronic medical records (*n* = 47; 25%) and various registries (*n* = 34; 18%). Some studies consulted more than one data source. For a detailed list of data sources, see Table 2. Studies conducted in Canada used hospital-based and government-based databases.

**Table 2:** Data sources used to assess and review injuries of interest.

Data source	*n*, %
Electronic medical records	47, 24.9%
Patient files, logs, presentations, in-patient and outpatient data, chart reviews, clinical/triage/call notes and reports, discharge summaries, anesthetic charts	42, 22.2%
Registries (i.e., trauma, emergency department, health record, clinical data, burn)	34, 18.0%
Patient admissions	32, 16.9%
Database review (i.e., hospitals, daily death certificates, trauma admissions, emergency department, patient, poisonings, referrals)	26, 13.8%
Governmental, police, or non-profit organization	14, 7.4%
Procedure room/theater/surgery lists	10, 5.3%
Imaging (e.g., CT, radiographs)	9, 4.2%
Google trends/Map Quest/Microsoft Bing services	5, 2.6%
Online survey	4, 2.1%
Publicly available data	3, 1.6%
Other: trauma activations, ambulance activations, phone counseling sessions, prehospital records, consult data, trauma handover list, autopsies, triage register, helicopter emergency requests	11, 5.8%
Media reports	1*

*Negligible percentage.

For an overview of gray literature included in this review, see Table 3.

**Table 3:** Gray literature results.

Injury categories	*n*, %	Type of source(s)	Region(s)
Poisoning	15, 36.6%	News, organization, academic, government	United States, United Kingdom (United Kingdom), Canada, India, France
Motor vehicle collisions	11, 26.8%	News, organization, public health, government, non-profit	United States, United Kingdom, Canada, Australia, India, Switzerland, New Zealand
Drowning	5, 12.2%	News, academic, government, non-profit	United States, Canada, New Zealand
Trampoline	4, 10.0%	News, government, non-profit	United States, Canada, United Kingdom
Unintentional injury	3, 7.3%	Organization, academic, public health	United States of America (United States), Australia
Cycling	1, 2.4%	Non-profit	United States
Orthopedic	1, 2.4%	Academic	United States
Falls	1, 2.4%	Blog	United States
Burns/fire safety	2, 5.0%	News	Canada

### Place of injury

Forty-eight (13–60) studies reported the place where incidents (e.g., injuries, poisonings) occurred, with most (*n* = 32; 66%) indicating an increase in incidents within domestic settings (13, 14, 17, 19–21, 23–25, 28, 29, 32–35, 37, 38, 40, 41, 43, 44, 46–49, 51–53, 56–59), 18 of which reported significant increases occurring at home. Domestic injuries were reportedly a result of do-it-yourself and home improvement projects (e.g., use of power tools, ladders, etc.), gardening, scalds, intoxications, exposures to household/cleaning products, and kitchen-related tasks. Only one study reported a decrease in domestic injuries during 2020 compared to the same periods in 2017–2019 (16). One study based in Ireland reported an increase in domestic injuries during the initial two-week lockdown period, followed by a decrease in the number of domestic injuries over the following 2 months (26). Five studies noted a decrease in injuries occurring outdoors and at school/daycare (36, 45, 58–60), four of which were significant decreases. Two studies based in the United Kingdom indicated that during the lockdown period, the majority of injuries occurred in the home or garden (31, 39). Interestingly, two studies reported an increase in injuries occurring outdoors or in public spaces during the lockdown (15, 24). Lastly, six studies reported no significant change in domestic injuries during the COVID-19 period (22, 27, 42, 50, 54, 55). The gray literature (*n* = 1) also indicated that there was an increase in emergency department visits for unintentional injuries occurring within the home (resulting from do-it-yourself projects).

### Unintentional injuries

Thirteen articles reported decreases in unintentional injuries (61–73), with four observing a significant decline during the pandemic period (65, 70, 72, 73). A total of five articles noted an increase in unintentional injuries overall (74–78) when comparing the lockdown period to the same time period in previous years, though none were significant. One research team in South Korea noted they observed a significant decrease in unintentional injuries resulting in facial trauma, but increases in hand trauma when compared to the same period in 2019 (59). Lastly, two studies indicated no differences in unintentional injuries during the pandemic period compared to pre-pandemic periods (79, 80). Gray literature (*n* = 3) reported increases in death rates as a result of unintentional injury (81–83). See Table 4 for an overview of injury results from both scientific evidence and gray literature.

**Table 4:** Injury results during COVID-19 from scientific evidence and gray literature.

	Scientific evidence		Gray literature
Injury category	Canada	Other countries	
Drowning	*n* = 1 (84) - Canada and United States based study - Beach drownings in the Great Lakes region significantly higher in 2020 than pre-COVID - Statistically significant increase in drownings for young males (< 20 years) during 2020 than pre-COVID		*n* = 5 (85–89) - Reports of increased drownings in open-water and an increase in pediatric drownings - Water Safety New Zealand report found that no fatal drownings had occurred during the first 4 weeks of the lockdown period.
Poisoning	*n* = 2 (65, 90) One study reported 61% decrease in pediatric poisonings during the COVID-19 pandemic Another study reported 40% increase in poisonings related to hand sanitizers and disinfectants shortly following the declaration of a pandemic	*n* = 8 (20, 33, 37, 77, 80, 91–93) Seven studies indicated increases in poisoning occurrences during the lockdown compared to previous periods. Reports of exposures to sanitizer, cleaners/disinfectants, algaecides, bleaches, chloride vapors, fertilizers, glues, silica gel, paints/varnish, batteries, household products, and essential oils increased. One study indicated a decrease in calls concerning exposures to medications, but an increase in exposures to household/cleaning products. United States-based study noted the increase of methanol ingestions, associated with swallowing alcohol-based hand sanitizers – in the United States, hand sanitizers are approved to only contain ethanol or isopropanol suggesting that methanol-based products may have been imported into the country. One study reported no change in poisonings.	*n* = 15 (94–107) Increases in calls to poison centers regarding hand sanitizers, cleaning products, inhalations, bleaches, disinfectants, mushroom exposures, youth, and older adults. A news article from United States outlined two case studies, one in which an individual cleaned her produce with a 10% bleach solution after buying her groceries, and the other about reported on a preschool child who ingested an unknown amount of hand sanitizer causing them to spend two nights in the hospital. Another news article from the United Kingdom stated that 5,800 people were admitted to the hospital as a result of false information they received on social media, and approximately 800 may have died from ingesting methanol or alcohol-based cleaning products. One article identified the harms of turning to homemade remedies that were not proven to protect against COVID-19.
Foreign body ingestion		*n* = 2 (32, 57) Reports of significant increases in foreign body ingestions among children, such as swallowing magnets, beads, plastic, marbles, coins, screws, and batteries from toys.	
Trampoline		*n* = 6 (16, 26, 45, 108–110) Four studies observed increases (three of which were significant) in trampoline injuries. One indicated a non-significant decrease, and one saw no significant differences between the time periods.	*n* = 4 (111–114) All noted increases in the proportion of injuries resulting from trampolines.
Lacerations		*n* = 18 (27) Studies reported on lacerations and/or animal/human bites during the COVID-19 pandemic lockdowns. Ten indicated increases in lacerations from sharp objects or animal bites (e.g., snakes and dogs). Five studies noted decreases in lacerations or bites. India-based study reporting a decrease in bull-gore injuries. One United Kingdom-based study noted no significant differences in animal bites during the lockdown period. Three studies that only investigated injuries during the pandemic period (without comparing to previous years) based in the United States, United Kingdom, and Iran noted that animal bites and injuries caused by sharp objects occurred during lockdown.	
Crush/Trap		*n* = 6 (27, 42, 76, 115–117) No significant difference between pre-pandemic and lockdown periods or a decrease between the two time points.	
Occupational		*n* = 25 (13, 23, 25, 29, 30, 35, 36, 41, 46, 50, 52–54, 58, 62, 118–127) 14 indicated decreases, five of which decreased significantly. Of the studies that noted type of work (*n* = 3), decreases were found in work including labor, mining, carpentry, and agricultural fieldwork. Another study explained that the decrease in worksite injuries resulted from a reduction in falls. One study noted an increase, on a relative scale, in violence-related injuries at work. Four studies reported increases in work-related injuries; however, this change was non-significant in two of the studies. Of the studies that noted type of work (*n* = 2), increases were noted in machine-related work and non-machine related work, and one industrial accident. One study also observed an increase in injuries in young workers in 2020 compared to 2019. An additional four studies reported no significant differences in occupational injuries between pre-pandemic and pandemic periods; however, one did note a decrease in injuries sustained while traveling to work. Type of work in these studies, where specified (*n* = 1) were physical labor related. Three cross-sectional studies described injury rates during the COVID-19 period. One study, based in India, noted that farm work-related injuries were most common during this time (106), and another described sharp and blunt injuries as a result of labor trauma were the most common mechanism of occupational injury.	
Burns	*n* = 1 (65) In children aged 0 to 5 years, burns significantly decreased in 2020 when compared to the average in the 2015–2019 period from 22 to 10 (55% decrease).	*n* = 16 (19, 26, 29, 32, 57, 59, 76, 91, 128–135) Five reported significant increases in burn-related injuries with four reporting non-significant increases. Four studies observed a significant reduction in burn injuries during lockdown. One study, conducted in Israel, indicated that the number of burns among children ages 2–5 increased significantly, and resulted from scalds and one case of electrical burn and burn-related injuries among adults decreased, with the most common cause being fire. A study conducted in Turkey saw an increase in third-degree burns and a reduction in second degree burns during the initial lockdown period in 2020, compared to cases in the same period in 2018 and 2019. Five studies indicated no significant changes in burns across both time periods. Cross-sectional survey with adults in the United States (*n* = 2011); 26% of households reported having experienced an injury, and 15% experienced an ingestion; 28% experienced either, and 13% experienced both. Of these injuries, burns from hot objects were reported by 5% of respondents and scalds by 4%.	*n* = 2 (136, 137) Based in Canada; 13% increase in fire-related calls over the COVID-19 pandemic, increases in significant fires, and multiple fatal fires from October 2020–January 2021.
Sports and recreation		*n* = 65 (13, 14, 16, 21, 23–25, 27, 29, 30, 34–36, 38–41, 43, 45, 48, 50–52, 54, 58, 59, 62, 63, 65, 70, 75, 108–110, 115, 118–121, 123–126, 133, 138–158) Majority (*n* = 54) indicated decreases in injuries resulting from sport, recreation, or leisure – including playing sports, running, and playground injuries – 28 of these reductions were significant. Four indicated increases in sport-related injuries during the pandemic, though none were significant, and three reported no significant changes over the two time periods. Two USA-based studies investigated running behaviors and injuries during the COVID-19 pandemic; one noted running-related injury risk was higher during the pandemic for lower extremity overuse injuries; the other noted prior history or injury was associated with new running-related injuries but there were no significant changes related to the running-related injuries and the pandemic. One study indicated an increase in indoor recreation injuries but a decrease in gym-related and soccer injuries. Another noted an increase in personal exercise injuries and decrease in injuries resulting from sports. One study in Iran studied emergency department admissions during their lockdown period and indicated that sports-related injuries occurred in 2% of patients (*n* = 13).	
Pedestrian		*n* = 20 (18, 19, 29, 50, 54, 66, 67, 71, 74, 116, 133, 140, 159–166) Decreases in injury incidence (*n* = 17); 10 were significant. One study indicated reduction was due to fewer individuals utilizing transit during the lockdown period. One study in Australia indicated an increase during the pandemic, acknowledging that numbers were small before and during restrictions. Another study analyzed data during the lockdown compared to two weeks before, and noted five injuries were pedestrian-related out of the 195 total patients seen during study period. One study saw no significant change in pedestrian injuries.	
Non-Motorized Vehicles (i.e., cycling, two-wheelers, rollerblades, skateboards, and hoverboards)		*n* = 31 (16–19, 26, 29, 31, 34, 35, 39, 49, 50, 54, 65, 70, 108, 110, 116, 123, 133, 139, 153, 159, 162, 163, 167–172) Nine studies indicated decreases in non-motorized vehicle injuries, three of which were significant. Overall, 18 studies indicated increases in non-motorized vehicle injuries, of which 12 were significant. Four research teams noted that injuries resulting from non-motorized vehicles were main causes of injuries during the pandemic.	*n* = 1 (173) Increase in number of cycling collisions during pandemic lockdowns; more than a quarter of cycling fatalities were caused by hit-and-run collisions in 2020, and that cycling fatalities were almost equal on urban and rural roads.
Falls		*n* = 105 (14, 15, 18, 19, 21, 24, 27, 29–31, 34, 38–40, 42, 44, 47, 49–55, 57–59, 62, 63, 66, 69, 70, 74, 75, 79, 91, 108–110, 115, 117, 119–122, 124–128, 133, 139–142, 144, 146, 148, 151, 153–159, 161–163, 165, 168, 170, 171, 174–204) *n* = 26 studies reported falls were most common mechanism of injury, regardless of whether the proportion or absolute number of fall cases increased or decreased. Forty articles reported increases in falls compared to previous periods, and 43 studies reported that falls decreased. Nine studies indicated that proportion of falls remained relatively consistent over the study periods. Five studies indicated that falls from height decreased and falls from ground-level increased. Two studies reported falls from ground level decreased and falls from height increased. Two studies, based in the UK and Ireland, indicated that falls reduced at the beginning of the lockdown but began to increase as restrictions eased from May–August, 2020.	*n* = 1 (205) 20% increase in falls occurring in memory care communities.
Motor Vehicle Collisions (i.e., vehicles and motorcycles)		*n* = 134 (13–19, 23, 24, 27–29, 34, 35, 38, 41–45, 47–50, 53–56, 59, 60, 62–71, 74–76, 78, 79, 108–110, 117, 118, 121–128, 133, 139–142, 144–148, 151–154, 156–166, 168, 170, 171, 174, 176, 178–183, 185–194, 196–199, 201, 204, 206–229) Regardless of whether MVCs increased or decreased, they remained one of the most common mechanisms of injury throughout the pandemic. Total of *n* = 114 indicated that MVCs decreased during lockdown periods compared to pre-pandemic periods. Interestingly, five of these studies – based in Russia, Israel, Turkey, the United States, and Nepal – observed that while MVCs decreased, the severity of the collisions worsened. Sedain and Pant (224): burden of MVCs remained high during the lockdown period, and although reduction in MVCs, the fatalities and injuries resulting from MVCs remained high. Qureshi et al. (222): significant reduction in MVCs resulting in minor to no injuries, but no reduction in MVCs resulting in serious or fatal injuries during statewide lockdowns; and increase in MVCs resulting in minor or no injuries when mandatory lockdown policies expired but no change in those resulting in serious or fatal injuries. Iran-based study: analyzed emergency department referrals in two trauma centers from February 20–April 3, 2020 and found that 21.3% (*n* = 136) of the trauma cases during this period resulted from MVCs. Eleven studies reported no significant changes between periods. Total of *n* = 10 studies noted increases in MVCs during the COVID-19 period, two of which also reported an increase in severity of MVCs.	*n* = 9 (230–240) Decrease in number of MVCs, but an increase in the severity of injuries. Many of these articles cited excessive speed as the cause for the heightened severity of cases.

## Discussion

This scoping review highlights the early impacts of public health measures implemented to reduce the spread of COVID-19 on the trends and patterns of unintentional injuries in Canada, and globally. With the rapid spread of the virus and declaration of a pandemic, populations were confined to their homes, and sports, school, recreation, certain jobs, and non-essential travel were interrupted, resulting in changes in injury patterns. Studies included in this review were predominantly based in Europe, the United States, and Asia, and typically employed a retrospective review of electronic medical records, registries, and databases. Owing to very few (*n* = 3) studies conducted in Canada at the time of this review, a comparison of data sources between Canadian and international studies could not be done. However, it is of interest to note which types of health data sources were most commonly used during the pandemic.

Studies reported that injuries occurred more frequently among males than females, and the average age across studies was approximately 46 years old. This aligns with current research that states that males are more likely than females to sustain and/or die from injuries (241). Interestingly, this review showed similarities in general trends globally during lockdown periods when compared to previous time points. The impact of the public health measures related to COVID-19 in Canada showed an increase in: (a) domestic injuries (e.g., resulting from do-it-yourself projects or gardening); (b) poisonings resulting from cleaners, disinfectants, and other products; (c) non-motorized vehicle injuries; (d) lacerations (e.g., from sharp objects and bites); (e) drownings; (f) trampoline injuries; and, (g) foreign body ingestions. Decreases were reported in injuries resulting from MVCs, occupation, sport and recreation, pedestrian-related collisions, and crush/trap injuries. The impact of Canadian COVID-19 public health measured showed mixed results for a small number of other mechanisms of injury, for instance, burns and falls showed almost equal numbers of studies reporting increases and decreases. In this review, gray literature sources reported on eight different types of injuries, all reporting an increase in incidence. While the increase in the occurrence or severity of injuries were consistent with the scientific evidence for six injuries (i.e., injuries at home, drowning, trampoline-related injuries, poisonings, non- motorized vehicle injuries, and MVCs), the findings on the other two mechanisms of injury were inconsistent (i.e., falls, burns). This is interesting to note in that sources that were more easily accessible to the public reported inconsistent findings compared to peer-reviewed articles with supporting evidence. This can lead to misconceptions among the public and a lack of understanding of the true burden of injury during disaster or pandemic situations.

An additional shared impact of COVID-19 public health restrictions in many countries was an initial drop in trauma admissions and hospital and health center presentations at the outset of the pandemic (63, 128, 179). When delving deeper into the decreases in injury reported across the various mechanisms of injury, it is first important to note that public health measures implemented to reduce the spread of a previously unknown virus caused fear of contagion among populations, which may have impeded individuals’ willingness to attend healthcare facilities for treatment of any kind (74). Additionally, the avoidance of healthcare facilities, as well as mandates from governments to stay at home, likely also resulted in an underreporting of injuries that occurred during COVID-19 lockdown periods.

General decreases in sport, recreation, crush/trap, and pedestrian injuries were not surprising given the cessation of organized sport and recreational activities, reduction in road traffic, and the rise in working from home. The reduction in occupational injuries was also expected; however, this was context dependent, in that one study reported on an increase in occupational injuries resulting from farm work. This highlights that essential industries were more vulnerable to a continuing risk of injury during the lockdown.

Falls and MVCs were reported as the most common mechanisms of injury during the pandemic, regardless of whether they increased or decreased. This trend remained consistent in that, in Canada, the leading causes for injuries and injury deaths are falls and transport incidents (6). Increased time within domestic settings may have led to a higher incidence of falls requiring medical attention. One research team suggested that promoting social connectedness via remote activities, rather than in person, may be a longitudinal practice that can reduce the incidence of vehicle collisions and injuries (227). They also noted that another factor that may have contributed to the reduction in MVCs was the reduction of alcohol-impaired driving, and therefore emphasis could be placed on engaging in alcohol-related activities with peers within the home and virtually, rather than in-person (227). The creation of other solutions to reduce the incidence of vehicle collisions and injuries, such as the development of more robust public transport system has also been suggested (227). Although, the frequency of MVCs was reported to decrease during COVID-19 lockdowns, an increase in the severity of injuries related to MVCs was reported by many countries. The increase in severity of MVCs was of interest, suggesting that individuals who were driving during COVID-19 lockdowns were more likely to engage in risk-taking behavior (e.g., speeding, racing) causing severe or fatal MVCs compared to before the lockdown (211).

The incidence of burns that occurred during the pandemic showed a similar trend to MVCs - while burns decreased, the incidence of more severe burns increased. In particular, one study team observed increased severity of burns among children (131). This may have been due to children spending more time at home, in conjunction with parents working from home and thus managing multiple responsibilities, which may have impacted their ability to consistently supervise children. Burns due to scalds were reported frequently during the pandemic, suggesting that children may have been reaching for hot surfaces or liquids that were unattended. Further, increased exposure to animals (also possibly unsupervised) during the lockdown may partly explain the surge in animal bites reported.

Only one study investigated the incidence of drownings during the COVID-19 pandemic, reporting a significant increase during the lockdown period (84). The authors suggested that the increase in beach visits may have been driven by the closing of public pools, and the closure of facilities providing swimming lessons. In addition, they note the presence of large crowds at beaches may have confirmed pre-existing biases that the beaches and conditions were safe, despite safety signage or the absence of a lifeguard (84). Further, the researchers explain that these biases are most often observed among young males, who are more susceptible to group think and pleasure seeking at the sacrifice of safety (84). Their results showed that there was a statistically significant increase in the number of young males (< 20 years old) who drowned in the Great Lakes during the pandemic (84).

The increase in reported poisonings is critical to analyze in that many countries experienced a high volume of calls or presentations related to ingestions of household cleaners, disinfectants, hand sanitizer, glue, batteries, and other household products. Researchers note that the increased time at home resulted in more frequent exposures to these products, especially for children, and that limited availability of certain cleaning products during the outset of the pandemic may have led to inappropriate mixing of other products, resulting in adverse outcomes (65, 90, 92). These findings suggest that efforts should be focused on increasing public awareness regarding ingestion or inhalation of household products, as well as checking that the products they are using have been approved by their country’s health authority (93). Further, children using hand sanitizers should be supervised and all products should be kept out of their reach, which is of particular concern when they are confined to the home environment (93), and the use of hand sanitizer was highly encouraged as part of the pandemic public messaging.

From this review, it is evident that public health policies can have a significant impact on mechanisms of unintentional injury and therefore targeted efforts should be made toward prevention and injury surveillance, particularly during lockdown or physical distancing periods. For example, an increase in injuries related to trampoline and non-motorized vehicles highlights the importance of targeted efforts to enhance public knowledge and behaviors related to informal sport activities. Further, examining the burden of unintentional injury and most common mechanisms of injury during lockdown periods allows healthcare professionals, researchers, and policymakers to understand gaps in healthcare services and responses with respect to preventing and treating these injuries. Thus, this review also aimed to identify how health data sources continued to be collected during the pandemic, and how similar methods might be implemented and utilized to assess injury patterns in Canadian systems in future events.

### Limitations

While this review provides a comprehensive overview of unintentional injuries during COVID-19 restrictions, there are limitations to note. First, the database searches included results from December 2019–July 2021 and newer studies have likely been published that are not included in this review, particularly from a Canadian perspective. These studies could provide additional insights into injury patterns in Canada, which would allow for comparison to global trends. Further, the studies included in this review compared COVID-19 periods to pre-lockdown phases; however, few comparisons were made to periods when restrictions were lifted. These outcomes would be of particular interest given that a few studies reported a “rebound” to previous injury levels when restrictions eased. As restrictions eased populations were permitted to spend more time outdoors engaging in distanced activities, and return to other activities of daily living, which may have led to an increase in injuries. Future research could investigate the pattern of injury trends following the relaxing of COVID-19 public health measures.

## Conclusion

This scoping review provides an updated and comprehensive summary of information on injury epidemiology during COVID-19 in Canada and across the globe, and reports on the early changes in injury patterns with regard to location, type, and severity of injuries during COVID-19 compared to before the pandemic period. The data show important injury trends that may require additional monitoring over the next few years as we return to pre-pandemic levels of engagement in community and workplace participation.

In addition, this review identifies key target areas to guide policy making and capacity planning, as well as public education. Although populations may have experienced fatigue related to health and safety messaging given the surplus of COVID-19 information disseminated during lockdown, it is important to also provide consistent education and reminders related to safety at home and during activities of daily living. The insights generated from this review can be used to inform interventions and policies to identify system gaps and reduce injury in populations, worldwide.

## Author contributions

SK: Conceptualization, Formal analysis, Investigation, Methodology, Writing – original draft, Writing – review & editing. SS: Conceptualization, Data curation, Formal analysis, Methodology, Writing – original draft, Writing – review & editing. OR: Formal analysis, Investigation, Writing – original draft, Writing – review & editing. WT: Conceptualization, Methodology, Project administration, Supervision, Writing – original draft, Writing – review & editing. SM: Conceptualization, Methodology, Project administration, Supervision, Writing – original draft, Writing – review & editing. IP: Conceptualization, Methodology, Project administration, Supervision, Writing – original draft, Writing – review & editing.
