# Supplementary material for: What was the impact of COVID-19 restrictions on unintentional injuries, in Canada and globally? A scoping review investigating how lockdown measures impacted the global burden of unintentional injury
Source: Front Public Health. 2024 Jun 3;12:1385452. doi: 10.3389/fpubh.2024.1385452 (PMC11180821; doi:10.3389/fpubh.2024.1385452)
Supplement: Supplementary file 1 [file Data_Sheet_1.pdf]

Appendix A: Scoping Review Search Strategy

COVID Injury Search Report

Search Structure

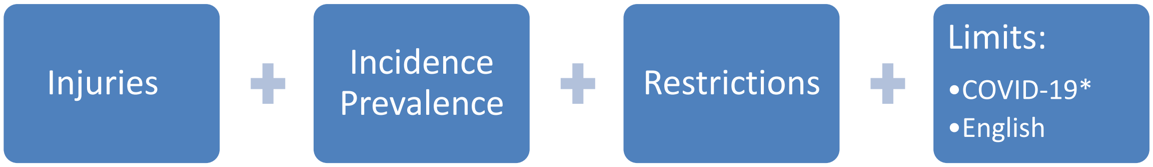

*Wolters Kluwer COVID 19 Search Filter used: https://tools.ovid.com/coronavirus/

Search Results

Database (Platform)	Date Searched	Number of results
MEDLINE (Ovid)	July 22, 2021	886
Embase (Ovid)	July 22, 2021	1132
Web of Science Core Collection	July 22, 2021	982
SPORTDiscus (EBSCO)	July 22, 2021	41
Total from database searches	---	3041

Search Strategies

MEDLINE

Ovid MEDLINE(R) and Epub Ahead of Print, In-Process, In-Data-Review & Other Non-Indexed Citations, Daily and Versions(R) <1946 to July 21, 2021>

1 "wounds and injuries"/ or exp abdominal injuries/ or accidental injuries/ or amputation, traumatic/ or exp arm injuries/ or asphyxia/ or athletic injuries/ or exp back injuries/ or exp barotrauma/ or exp birth injuries/ or exp "bites and stings"/ or exp burns/ or exp cold injury/ or contrecoup injury/ or exp crush injuries/ or exp joint dislocations/ or exp drowning/ or exp electric injuries/ or esophageal perforation/ or "extravasation of diagnostic and therapeutic materials"/ or exp foreign bodies/ or exp fractures, bone/ or fractures, cartilage/ or exp frostbite/ or exp hand injuries/ or exp heat stress disorders/ or exp hip injuries/ or lacerations/ or exp leg injuries/ or microtrauma, physical/ or exp multiple trauma/ or nasal septal perforation/ or exp neck injuries/ or occupational injuries/ or exp radiation injuries/ or reinjuries/ or retropneumoperitoneum/ or exp rupture/ or exp shock, traumatic/ or exp shoulder injuries/ or exp soft tissue injuries/ or exp spinal cord injuries/ or exp "sprains and strains"/ or exp tendon injuries/ or exp thoracic injuries/ or exp tooth injuries/ or exp trauma, nervous system/ or tympanic membrane perforation/ or vascular system injuries/ or exp wounds, nonpenetrating/ or exp wounds, penetrating/ 936409

2 accidents/ or exp accident prevention/ or accidental falls/ or accidents, aviation/ or exp biohazard release/ or exp chemical hazard release/ or accidents, home/ or accidents, occupational/ or accidents, traffic/ or exp radioactive hazard release/ 195098

3 (injur* or accident* or burn? or fire? or fall? or poison* or drown* or fracture? or trauma or collision? or crashes or suffocat*).tw,kf. 1655590

4 or/1-3 2147136

5 incidence/ or prevalence/ or Epidemiology/ 577470

6 (incidence or prevelan* or epidemiolog* or trend? or pattern? or occurrence or change?).tw,kf. 5760350

7 (increase? or decrease? or reduc* or effect?).tw,kf. 11136589

8 or/5-7 13868560

9 4 and 8 1103617

10 Quarantine/ 4965

11 (lockdown? or restriction? or quarantine? or shelter or social distancing or physical distancing or confinement).tw,kf. 253149

12 (stay* adj2 home).tw,kw. 2674

13 or/10-12 256900

14 4 and 8 and 13 9714

15 limit 14 to covid 19 916

16 limit 15 to english language 886

Embase

Embase <1974 to 2021 July 21>

1 injury/ or exp abdominal injury/ or exp abrasion/ or accidental injury/ or automutilation/ or exp avulsion injury/ or exp barotrauma/ or exp birth injury/ or exp "bites and stings"/ or exp blood vessel injury/ or exp blunt trauma/ or breast injury/ or exp burn/ or exp chemical injury/ or exp childhood injury/ or exp concussion/ or contrecoup injury/ or exp contusion/ or crush trauma/ or exp drowning/ or exp electric injury/ or exp erosion/ or exp foreign body/ or exp "head and neck injury"/ or exp heart injury/ or immune injury/ or immune mediated injury/ or exp injury scale/ or injury severity/ or exp limb injury/ or membrane damage/ or microtrauma/ or multiple trauma/ or exp musculoskeletal injury/ or exp nervous system injury/ or organ injury/ or exp pelvis injury/ or exp perforation/ or prenatal injury/ or exp psychotrauma/ or exp radiation injury/ or exp reperfusion injury/ or exp respiratory tract injury/ or exp rupture/ or seatbelt injury/ or exp skin injury/ or exp soft tissue injury/ or sport injury/ or strangulation/ or surgical injury/ or exp thermal injury/ or exp thorax injury/ or tissue injury/ or exp tooth injury/ or traumatic amputation/ or traumatic hematoma/ or exp traumatic shock/ or trench foot/ or exp urogenital tract injury/ or volutrauma/ or exp wound/ 2306353

2 accident/ or aircraft accident/ or exp biological accident/ or exp chemical accident/ or destruction/ or exp disaster/ or electric accident/ or electrocution/ or explosion/ or falling/ or home accident/ or exp nuclear accident/ or exp occupational accident/ or radiation accident/ or structure collapse/ or traffic accident/ 207001

3 accident prevention/ 15828

4 (injur* or accident* or burn? or fire? or fall? or poison* or drown* or fracture? or trauma or collision? or crashes or suffocat*).tw,kw. 2025852

5 or/1-4 3300591

6 incidence/ 460767

7 prevalence/ or epidemiology/ 992500

8 (incidence or prevelan* or epidemiolog* or trend? or pattern? or occurrence or change?).tw,kw. 7335228

9 (increase? or decrease? or reduc* or effect?).tw,kw. 13915670

10 or/6-9 17432385

11 5 and 10 1877547

12 home quarantine/ 1842

13 (lockdown? or restriction? or quarantine? or shelter or social distancing or physical distancing or confinement).tw,kw. 294670

14 (stay* adj2 home).tw,kw. 3203

15 or/12-14 298272

16 11 and 15 16475

17 limit 16 to covid-19 1153

18 limit 17 to english language 1132

Web of Science Core Collection

17
#8 AND #15 and **English** (Languages)
Edit
Add to Search
982

16
#8 AND #15
Edit
Add to Search
1,016

15
#9 OR #10 OR #11 OR #12 OR #13 OR #14
Edit
Add to Search
164,875

14
TS=((covid or covid19 or covid-19) and pandemic*)

Edit
Add to Search
72,532
13
TS=((nouveau NEAR/2 CoV) or (nouveau NEAR/2 nCoV) or (nouveau NEAR/2 covid) or (nouveau NEAR/2 coronavirus*) or (nouveau NEAR/2 corona virus) or (nouveau NEAR/2 Pandemi*))
Edit
Add to Search
2
12
TS=((new NEAR/2 CoV) or (new NEAR/2 nCoV) or (new NEAR/2 covid) or (new NEAR/2 coronavirus*) or (new NEAR/2 corona virus) or (new NEAR/2 Pandemi*))
Edit
Add to Search
5,381
11
TS=((novel NEAR/2 CoV) or (novel NEAR/2 nCoV) or (novel NEAR/2 covid) or (novel NEAR/2 coronavirus*) or (novel NEAR/2 corona virus) or (novel NEAR/2 Pandemi*))
Edit
Add to Search
10,996
10
TS=(2019-ncov or ncov19 or ncov-19 or 2019-novel CoV or sars-cov2 or sars-cov-2 or sarscov2 or sarscov-2 or Sars-coronavirus2 or Sars-coronavirus-2 or SARS-like coronavirus* or coronavirus-19 or covid19 or covid-19 or covid 2019)
Edit
Add to Search
153,911
9
TS=(coronavirus* or corona virus* or OC43 or NL63 OR 229E or HKU1 or HCoV* or nvoc* or covid* or sars-cov* or sarscov* or sars-coronavirus* or severe acute respiratory syndrome coronavirus*) Limited to date= 2019-01-01 to 2021-12-31
Edit
Add to Search
162,859
8
#1 AND #4 AND #7
Edit
Add to Search
22,231
7
#5 OR #6
Edit
Add to Search
585,242
6
TS=(stay* NEAR/2 home)
Edit
Add to Search
4,031
5
TS=(lockdown$ or restriction$ or quarantine$ or shelter or social distancing or physical distancing or confinement)

Edit
Add to Search
582,147

4
#2 OR #3
Edit
Add to Search
25,139,956

3
TS=(increase$ or decrease$ or reduc* or effect$)
Edit
Add to Search
20,212,996

2
TS=(incidence or prevelan* or epidemiolog* or trend$ or pattern$ or occurrence or change$)
Edit
Add to Search
10,048,332

1
TS=(injur* or accident* or burn$ or fire$ or fall$ or poison* or drown* or fracture$ or trauma or collision$ or crashes or suffocat*)
Edit
Add to Search
2,982,434

SPORTDiscus

#	Query	Limiters/Expanders	Last Run Via
S19	S4 AND S8 AND S11 AND S18	Search modes - Boolean/Phrase	Interface - EBSCOhost Research D<br>Search Screen - Advanced Search<br>Database - SPORTDiscus
S18	S12 OR S13 OR S14 OR S15 OR S16 OR S17	Search modes - Boolean/Phrase	Interface - EBSCOhost Research D<br>Search Screen - Advanced Search<br>Database - SPORTDiscus
S17	TI (((covid or covid19 or covid-19) and pandemic*)) OR AB (((covid or covid19 or covid-19) and pandemic*)) OR KW (((covid or covid19 or covid-19) and pandemic*))	Search modes - Boolean/Phrase	Interface - EBSCOhost Research D<br>Search Screen - Advanced Search<br>Database - SPORTDiscus
S16	TI (((nouveau N2 CoV) or (nouveau N2 nCoV) or (nouveau N2 covid) or (nouveau N2 coronavirus*) or (nouveau N2 corona virus) or (nouveau N2 Pandemi*))) OR AB (((nouveau N2 CoV) or (nouveau N2 nCoV) or (nouveau N2 covid) or (nouveau N2 coronavirus*) or (nouveau N2 corona virus) or (nouveau N2 Pandemi*))) OR KW (((nouveau N2 CoV) or (nouveau N2	Search modes - Boolean/Phrase	Interface - EBSCOhost Research D<br>Search Screen - Advanced Search<br>Database - SPORTDiscus

	nCoV) or (nouveau N2 covid) or (nouveau N2 coronavirus*) or (nouveau N2 corona virus) or (nouveau N2 Pandemi*)))		
S15	TI (((new N2 CoV) or (new N2 nCoV) or (new N2 covid) or (new N2 coronavirus*) or (new N2 corona virus) or (new N2 Pandemi*))) OR AB (((new N2 CoV) or (new N2 nCoV) or (new N2 covid) or (new N2 coronavirus*) or (new N2 corona virus) or (new N2 Pandemi*))) OR KW (((new N2 CoV) or (new N2 nCoV) or (new N2 covid) or (new N2 coronavirus*) or (new N2 corona virus) or (new N2 Pandemi*)))	Search modes - Boolean/Phrase	Interface - EBSCOhost Research D<br>Search Screen - Advanced Search<br>Database - SPORTDiscus
S14	TI (((novel N2 CoV) or (novel N2 nCoV) or (novel N2 covid) or (novel N2 coronavirus*) or (novel N2 corona virus) or (novel N2 Pandemi*))) OR AB (((novel N2 CoV) or (novel N2 nCoV) or (novel N2 covid) or (novel N2 coronavirus*) or (novel N2 corona virus) or (novel N2 Pandemi*))) OR KW (((novel N2 CoV) or (novel N2 nCoV) or (novel N2 covid) or (novel N2 coronavirus*) or (novel N2 corona virus) or (novel N2 Pandemi*)))	Search modes - Boolean/Phrase	Interface - EBSCOhost Research D<br>Search Screen - Advanced Search<br>Database - SPORTDiscus
S13	(DE "COVID-19" OR DE "COVID-19 pandemic") OR TI ((2019-ncov or ncov19 or ncov-19 or 2019-novel CoV or sars-cov2 or sars-cov-2 or sarscov2 or sarscov-2 or Sars-coronavirus2 or Sars-coronavirus-2 or SARS-like coronavirus* or coronavirus-19 or covid19 or covid-19 or covid 2019)) OR AB ((2019-ncov or ncov19 or ncov-19 or 2019-novel CoV or sars-cov2 or sars-cov-2 or sarscov2 or sarscov-2 or Sars-coronavirus2 or Sars-coronavirus-2 or SARS-like coronavirus* or coronavirus-19 or covid19 or covid-19 or covid 2019)) OR KW ((2019-ncov or ncov19 or ncov-19 or 2019-novel CoV or sars-cov2 or sars-cov-2 or sarscov2 or sarscov-2 or Sars-coronavirus2 or Sars-coronavirus-2 or SARS-like coronavirus* or	Search modes - Boolean/Phrase	Interface - EBSCOhost Research D<br>Search Screen - Advanced Search<br>Database - SPORTDiscus

	coronavirus-19 or covid19 or covid-19 or covid 2019))		
S12	TI ((coronavirus* or corona virus* or OC43 or NL63 OR 229E or HKU1 or HCoV* or nvoc* or covid* or sars-cov* or sarscov* or sars-coronavirus* or severe acute respiratory syndrome coronavirus*)) OR AB ((coronavirus* or corona virus* or OC43 or NL63 OR 229E or HKU1 or HCoV* or nvoc* or covid* or sars-cov* or sarscov* or sars-coronavirus* or severe acute respiratory syndrome coronavirus*)) OR KW ((coronavirus* or corona virus* or OC43 or NL63 OR 229E or HKU1 or HCoV* or nvoc* or covid* or sars-cov* or sarscov* or sars-coronavirus* or severe acute respiratory syndrome coronavirus*))	Limiters - Published Date: 20190101-20211231 Search modes - Boolean/Phrase	Interface - EBSCOhost Research D Search Screen - Advanced Search Database - SPORTDiscus
S11	S9 OR S10	Search modes - Boolean/Phrase	Interface - EBSCOhost Research D Search Screen - Advanced Search Database - SPORTDiscus
S10	TI (stay* N2 home) OR AB (stay* N2 home) OR KW (stay* N2 home)	Search modes - Boolean/Phrase	Interface - EBSCOhost Research D Search Screen - Advanced Search Database - SPORTDiscus
S9	(DE "STAY-at-home orders") OR TI ((lockdown# or restriction# or quarantine# or shelter or social distancing or physical distancing or confinement)) OR AB ((lockdown# or restriction# or quarantine# or shelter or social distancing or physical distancing or confinement)) OR KW ((lockdown# or restriction# or quarantine# or shelter or social distancing or physical distancing or confinement))	Search modes - Boolean/Phrase	Interface - EBSCOhost Research D Search Screen - Advanced Search Database - SPORTDiscus
S8	S5 OR S6 OR S7	Search modes - Boolean/Phrase	Interface - EBSCOhost Research D Search Screen - Advanced Search Database - SPORTDiscus
S7	TI ((increase# or decrease# or reduc* or effect#)) OR AB ((increase# or decrease# or reduc* or effect#)) OR KW ((increase# or decrease# or reduc* or effect#))	Search modes - Boolean/Phrase	Interface - EBSCOhost Research D Search Screen - Advanced Search Database - SPORTDiscus
S6	TI ((incidence or prevelan* or epidemiolog* or trend# or pattern# or occurrence or change#)) OR AB ((incidence or prevelan* or	Search modes - Boolean/Phrase	Interface - EBSCOhost Research D Search Screen - Advanced Search Database - SPORTDiscus

	epidemiolog* or trend# or pattern# or occurrence or change#)) OR KW ((incidence or prevelan* or epidemiolog* or trend# or pattern# or occurrence or change#))		
S5	DE "DISEASE prevalence" OR DE "EPIDEMIOLOGY"	Search modes - Boolean/Phrase	Interface - EBSCOhost Research D<br>Search Screen - Advanced Search<br>Database - SPORTDiscus
S4	S1 OR S2 OR S3	Search modes - Boolean/Phrase	Interface - EBSCOhost Research D<br>Search Screen - Advanced Search<br>Database - SPORTDiscus
S3	TI ((injur* or accident* or burn# or fire# or fall# or poison* or drown* or fracture# or trauma or collision# or crashes or suffocat*)) OR AB ((injur* or accident* or burn# or fire# or fall# or poison* or drown* or fracture# or trauma or collision# or crashes or suffocat*)) OR KW ((injur* or accident* or burn# or fire# or fall# or poison* or drown* or fracture# or trauma or collision# or crashes or suffocat*))	Search modes - Boolean/Phrase	Interface - EBSCOhost Research D<br>Search Screen - Advanced Search<br>Database - SPORTDiscus
S2	DE "ACCIDENTS" OR DE "ACCIDENTAL falls" OR DE "CAVING accidents" OR DE "CRASH injuries" OR DE "DROWNING" OR DE "HUNTING accidents" OR DE "ICE accidents" OR DE "MARINE accidents" OR DE "MEDICAL emergencies" OR DE "MOUNTAINEERING accidents" OR DE "OVERUSE injuries" OR DE "SKIING accidents" OR DE "SPORTS accidents"	Search modes - Boolean/Phrase	Interface - EBSCOhost Research D<br>Search Screen - Advanced Search<br>Database - SPORTDiscus
S1	DE "WOUNDS & injuries" OR DE "BACKPACKING injuries" OR DE "BLUNT trauma" OR DE "BURNS & scalds" OR DE "CAMPING injuries" OR DE "CHRONIC wounds & injuries" OR DE "CRASH injuries" OR DE "DANCING injuries" OR DE "DECOMPRESSION sickness" OR DE "DISABILITIES" OR DE "HEAD injuries" OR DE "JOINT dislocations" OR DE "MARTIAL arts injuries" OR DE "MOTORCYCLING injuries" OR DE "MOUNTAINEERING injuries" OR DE "NERVOUS system injuries"	Search modes - Boolean/Phrase	Interface - EBSCOhost Research D<br>Search Screen - Advanced Search<br>Database - SPORTDiscus

OR DE "ORGAN rupture" OR DE
"OVEREXERTION injuries" OR DE
"OVERUSE injuries" OR DE
"PENETRATING wounds" OR DE
"PHYSIOLOGIC strain" OR DE
"SOFT tissue injuries" OR DE
"SPORTS injuries" OR DE
"SUBLUXATION" OR DE
"WOUND care"

1

2
